# Why-Oh-Why? Dark Brooders Reduce Injurious Pecking, Though Are Still Not Widely Used in Commercial Rearing of Layer Pullets

**DOI:** 10.3390/ani12101276

**Published:** 2022-05-16

**Authors:** Janja Sirovnik, Anja B. Riber

**Affiliations:** 1 Department of Farm Animals and Veterinary Public Health, Institute of Animal Welfare Science, University of Veterinary Medicine, 1210 Vienna, Austria; 2 Department of Animal Science, Aarhus University, DK-8830 Tjele, Denmark; anja.riber@anis.au.dk

**Keywords:** chicken, laying hen, feather pecking, behaviour, fear, maternal care

## Abstract

**Simple Summary:**

Dark brooders mimic some aspects of maternal care, such as the provision of heat and a dark resting area. Dark brooders have a long-lasting reducing effect on injurious pecking, which improves feather cover and reduces mortality due to cannibalism into adulthood. The economic benefits include a reduction of costs for heating in the first weeks of life, and in adult layers, they improve the total egg production and may reduce floor egg laying. Despite the well-accepted knowledge of the positive effects of dark brooders on the prevention of injurious pecking, few farmers actually use them in their rearing facilities. In this paper, we review the effects of dark brooders on the welfare of pullets and layers and discuss the factors that likely contribute to their low usage in commercial systems, including the lack of commercially available brooder options and the size of the brooders, as well as the lack of information on the direct economic aspects.

**Abstract:**

Dark brooders, i.e., horizontal heating elements for chicks equipped with curtains, mimic some aspects of maternal care, such as the provision of heat and a dark area for chicks to rest. Thus, they can be considered as artificial passive replacements of a mother hen. Despite their advantages in animal welfare and the likely positive outcomes in production and economy, dark brooders are rarely used in commercial layer pullet facilities. The main positive effect on welfare is a reduction of injurious pecking during the rearing and laying periods, which results in improved feather cover and reduced skin injuries and mortality due to cannibalism. Other welfare benefits include improved rest in dark-brooded chicks and reduced fearfulness at all ages tested (i.e., from 4 to 26 weeks). The impact on production and economy is seen in a reduction of the energy costs in the first weeks of life due to radiant heating, as well as improved total egg production and reduced floor egg laying. The aim of this paper is to review the existing literature on the effects of dark brooders on injurious pecking and other welfare issues in layers, including speculations on the possible explanations for improved welfare. We also discuss the possible reasons for why dark brooders are not applied more commonly in commercial practice, including insufficient information on the economic aspects of using brooders and the lack of commercially available brooder options.

## 1. Introduction

Injurious pecking can be defined as abnormal behaviour characterised with severe pecking at the feathers and/or denuded areas of other birds [1,2,3]. Injurious pecking is prevalent in all housing systems (e.g., [4]) and can lead to high mortality. For example, one study reported 3.9% and 17% mortality in barn-housed and free-range layer flocks, respectively [4]. Furthermore, feather damage and skin wounds have been reported in more than half of the birds in 39% and 16% of organic flocks, respectively [5]. Skin wounds are also prevalent in cages [4]. Injurious pecking negatively affects pullet and layer welfare through pain and fear due to the forced removal of feathers and skin injuries [3,6], difficulties in maintaining body temperature [7] and secondary infections of the wounded areas [4]. Additionally, production traits, such as total egg production, flock mortality and uniformity, and feed conversion efficiency are also adversely affected by injurious pecking, reducing the farm profits (e.g., [8]). Injurious pecking thus creates hen welfare issues and economic problems. Outbreaks of injurious pecking are unpredictable and, once in progress, are difficult to control. Thus, measures should focus on preventing injurious pecking instead of treating it.

Researchers have investigated how to prevent and control injurious pecking for over a century [9], and yet, it remains one of the most severe welfare and economic problems in the layer industry. Beak trimming is a commonly used preventative measure of injurious pecking, but it raises ethical concerns related to the welfare of the animals due to induced acute and potential chronic pain [10,11]. Likewise, consumer demands for production systems allowing more natural behaviours are increasing in some parts of the world, e.g., in Europe, where it has led to the European Citizens’ Initiative ‘End the Cage Age’. It is well-known that managing laying hens with intact beaks is more demanding, especially in non-cage systems. Thus, with the move towards non-cage systems and birds with intact beaks, it becomes more urgent than ever to find measures to prevent or reduce the extent of injurious pecking. One such measure is rearing chicks with artificial replacements of mothers, i.e., dark brooders. The first full paper that showed the positive effects of dark brooders on the reduction of injurious pecking was published 16 years ago [12], and since then, other studies have confirmed the findings in small-scale settings [13] and on commercial farms [14]. Despite the well-accepted knowledge of the positive effects of dark brooders on the prevention of injurious pecking, research on the actual use of dark brooders in commercial systems is scarce. Through informal personal communication with pullet producers and their advisors in several European countries, we learnt that few pullet producers install brooders in their pullet rearing facilities, and most farmers that use brooders practice floor rearing. There may be various reasons for this.

The aim of this paper is twofold. First, we describe dark brooders and review the existing literature on the effects of dark brooders on injurious pecking and other welfare issues in pullets and laying hens, including speculations on the possible explanations for improved welfare. In the second part, we focus on the commercial use of dark brooders. We discuss the possible reasons for why dark brooders, as a preventive measure against injurious pecking, are not applied more commonly in commercial practice. We also highlight gaps in the knowledge on dark brooders that, if filled in, may help producers of commercial pullets with the transition to using brooders as the source of heating. As a proper economic study has not yet been conducted, we decided to review the impact of dark brooders on the economy by focusing on the known production effects, such as improved total egg laying and reduced floor laying of brooded layers, while highlighting the factors that still need to be studied.

## 2. Materials and Methods

The literature search was conducted in October 2021. We targeted the database Web of Science using the key terms “brooder”, “laying hen” and “behaviour”. Only publications written in English were included. Our initial search yielded 11 titles. The total number of titles was reduced to 9 after an initial review of the title and abstract. This was supplemented with one article under revision that we were informed about.

## 3. Dark Brooders Mimic Some Central Aspects of Maternal Care

**Figure 1:**
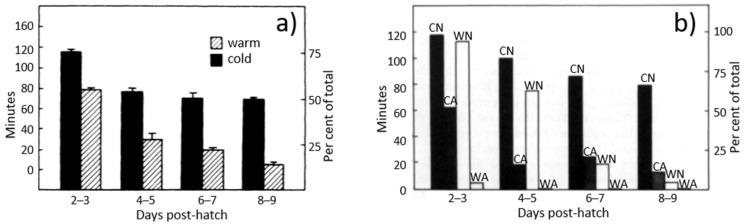
Mean time spent brooding by chicks when in a brood of five under either (**a**) the mother hen ^1^ (observation period: 180 min) or (**b**) the brooder ^2^ (observation period: 120 min) [15]. ^1^ ‘Warm’ is an air temperature of 28 ± 2 °C, and ‘cold’ is 19 ± 1 °C. Error bars equal ±1 standard error of the mean. ^2^ ‘N’: brooder was either heated to the hen’s body temperature of 41 ± 0.2 °C or ‘A’: left unheated and, thus, had the same temperature as the air. Air temperature was either ‘W’: 28 ± 2 °C or ‘C’: 19 ± 1 °C.

Although the chicken is a precocial species that does not depend on the mother to find feed and water, it is unable to regulate its own body temperature during the first approximately 10 days of life [15]. Under natural conditions, chicks receive warmth from the mother hen and spend a great proportion of time under her (Figure 1a). After the first days of life, chicks spend less time under the mother hen but stay with her for the first 5–12 weeks of age and will then gain their independence [16].

Natural brooding is not commercially viable. Hence, an artificial heating source in either the form of radiant (e.g., infrared light) or full-house heating is commonly provided to chickens under commercial rearing conditions [17]. Various welfare issues have been identified in chickens reared without maternal care, such as increased aggression [18] and a lack of spatial skills [19]. Chicks reared without a mother hen can also show increased levels of injurious pecking during adulthood [20,21], but no effect on injurious pecking has been shown during the early rearing period, e.g., at 7 days [22] or 42 days [23]. In addition, chickens reared without mother hens show increased levels of fearfulness as chicks, pullets and laying hens [22,23,24], which has been related to injurious pecking [20].

Instead of using mother hens, artificial passive models of a mother hen—dark brooders—have been introduced to commercial rearing farms. A study by Sherry [15], where various characteristics of a mother hen were tested using the complex model of a mother hen (i.e., brooder) by stuffing a sacrificed hen and equipping it with a heater, revealed that the heat provided by the brooder was sufficient to ensure that chicks rested under the brooder. Other properties of the brooder shared with a mother hen, such as colour, shape and texture, did not affect the chicks’ preference. Comparing the results of two trials, where the first trial recorded time spent under the mother hen (Figure 1a) and the other trial recorded time spent under artificial brooders (Figure 1b) during a 2-h observation period in the early afternoon, it was revealed that heated brooders were used to a similar extent as mother hens. Independently of the environmental temperature, chicks spent most of the time under the mother hens and heated brooders in the first 3 and 5 days of life, respectively. This suggests that chicks have a great need for this type of maternal care in the first 5 days of life independent of the environmental temperature. As the environmental temperature did not affect the use of the heated brooders in the first week of age, a lower environmental temperature may likely be used even for young chicks when brooders are provided. After day 5, the time spent under the mother hen and brooder during the 2-h observation period depended on the environmental temperature. If the environmental temperature was low, the use persisted into the second week of life, and if the environmental temperature was high, the use almost stopped after the first week of life.

Based on the study by Sherry [15], simple brooders equipped with fabric fringes on all sides and a heating source on top have been designed to provide chicks with a warm and dark resting space. The warmth under the brooder is ensured by mounting either electrical heat panels [12,14,25,26] or water-heated panels [13,27] inside the brooder. The temperature under dark brooders is usually lower than in the study by Sherry [15]. Typically, it is kept at approximately 30–34 °C for the first 2 days of the chicks’ lives and is then slowly reduced over 4 weeks, similar to the standard commercial practice for the ambient temperature when practicing whole-house heating. This is accomplished either by increasing the height of the brooders or by reducing the heat supply to the brooders. When brooders are used as an artificial heating source, the ambient temperature of the barn is kept cooler (e.g., at 24 °C in the first days and then at 20 °C [14,27] or 18 to 19 °C over the whole rearing period [12]), leading to reduced heating costs. Some farmers provide dark brooders without a heating source, i.e., only a dark area that allows chicks to gather under (personal communication, David Brass, The Lakes Free Range Egg Company, UK). In those brooders without a heating source, the temperature under the brooders is, as such, similar to the one outside the brooders but may be partly regulated through the chicks’ own heat production related to their metabolism. Thus, even if brooders provide no additional heating source, it may still be possible to create a difference in temperatures under the brooders and the rest of the facility, though this type of dark brooder likely has lower economic benefits. The study by Sherry [15] clearly indicated that chicks prefer heat-emitting brooders, as the use of unheated brooders was significantly lower. Future research is necessary to describe the use of nonheated brooders by the chicks and the related welfare and production effects.

Dark brooders have mainly been studied for the rearing of pullets, and the duration of their provision has differed greatly between and even within studies: 5 weeks [12], 7 weeks [13,27], 8 weeks [14] or 16 weeks [14]. Studies have been conducted on the effect of brooders on behaviour and welfare in slow-growing broilers [26] and fast-growing broilers [28], where dark brooders were provided for only 3 weeks. Gilani et al. [14] found no effect of the duration of brooder provision (8 vs. 16 weeks) on their behaviour and concluded that the main effect of dark brooders likely occurs before the birds are 8 weeks old, though the sample size was very small (i.e., one and two replicates per duration). As chicks seem to use brooders less after the first week of age (provided that the barn temperature is age-appropriate) [15], brooders may be offered for shorter periods than applied in studies so far, but the economic advantages are likely reduced due to the use of whole-barn heating. Further, the effect of such a short provision of brooders on welfare should be examined.

## 4. Welfare Improvements Gained by Rearing with Dark Brooders

**Figure 2:**
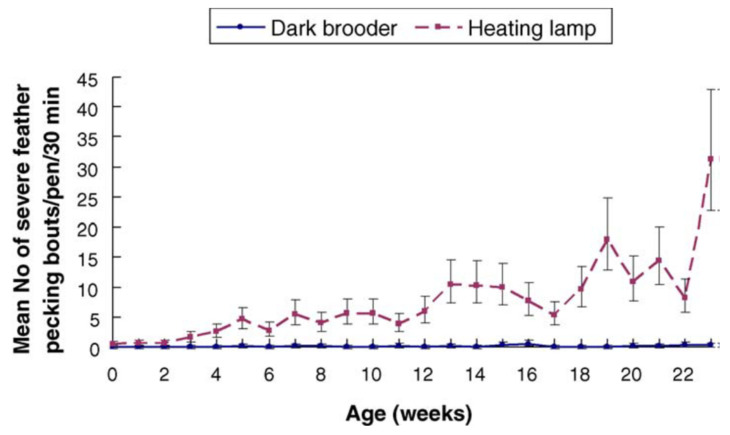
Mean number and SE of severe feather pecking bouts per pen per 30 min for the dark brooder pens and the heating lamp (i.e., non-brooded) pens [12].

Rearing pullets with dark brooders has long-lasting positive effects on the animals’ welfare, among other things, by preventing and reducing the prevalence of injurious pecking (Figure 2).

There are various types of injurious pecking [1]. Severe feather pecking is characterised by pulling at and/or the forceful removal of feathers [2] and may progress into cannibalism targeting the skin of the body [2,3]. Vent pecking and toe pecking are also forms of injurious pecking that may occur both dependently and independently of severe feather pecking [2,29,30]. Gentle feather pecking is not a form of injurious pecking, though it can sometimes lead to injurious pecking [31]. While pullets and layers reared without brooders express various levels of injurious pecking, the prevalence of severe feather pecking and cannibalism is highly reduced or even absent in pullets and layers reared with brooders either on commercial farms [14] or in experimental studies [12,27]. Already, during the first 4 days of life, a higher level of feather pecking has been observed in non-brooded (0.9%) compared to brooded (0.1%) layer chicks [27], but the study did not differentiate between gentle and severe feather pecking. As injurious pecking that starts during the first days of life is likely to continue throughout the rearing period, future research should investigate this further [32]. Differences in the prevalence of injurious pecking between brooded and non-brooded birds have been reflected in the level of plumage damage [12,13,14], skin injuries [12,13] and mortality [12,13]. Compared to non-brooded birds, the plumage conditions of brooded birds were improved continuously until the end of the respective studies (i.e., the longest study duration: 35 weeks of age [14]). The positive effect of brooders on the reduction of skin injuries was more pronounced in the egg production period (i.e., at 23 weeks of age [12] and 28 weeks of age [13]) than during rearing [13]. Fewer pullets and layers brooded during rearing died or were euthanised due to injurious pecking compared to non-brooded pullets and layers [12,13]. Jensen et al. [12] reported that, in flocks of beak-intact brown layers reared with dark brooders, only one individual died due to cannibalism during the entire experiment, which lasted until the birds reached 23 weeks of age, whereas a total of 24 (26.7%) individuals from five of the six non-brooded pens were either found dead or injured due to cannibalistic pecks. In the study by Riber and Guzman [13], 0.7% and 5.9% of dark-brooded birds died or were culled due to cannibalism by 16 and 28 weeks of age, respectively, as compared to 7.7% and 29.3% of non-brooded birds, and the study had to be terminated for ethical reasons. Until now, all studies of the effect of brooders on layers have used brown genotypes, i.e., it remains to be examined to what extent the effects of brooders also apply to white genotypes.

It remains to be determined why dark brooders have a suppressing impact on the development of injurious pecking. It has been suggested that the separation of active and inactive chicks in a physical space when rearing with dark brooders reduces the risk of misdirected pecks [25]. When brooders are provided, only a small percentage of birds can be found resting outside the brooders during the first days of life. In contrast, individuals found outside the brooders are mainly active [27]. By pecking at various objects, chicks learn to recognise food and dust bathing materials in the first 10 days of life [33,34,35,36]. Exploratory pecks at food and dust bathing/foraging materials may become misdirected towards inactive conspecifics, with the risk of down/feathers being recognised as foraging materials, and hence, a development for feather pecking may occur [36,37]. Since feather pecks are more often directed towards inactive than active conspecifics [38], separating active and inactive individuals may have a reducing effect on feather pecking. Indeed, non-brooded layer chicks seem to have an increased interest in the feathers of their conspecifics compared to brooded layer chicks, as a greater frequency of feather pecking has been observed in non-brooded birds both during the first 4 days of life and from the first week of life until adulthood [12,27]. In contrast, dark-brooded pullets explore the environment more compared to the non-brooded pullets at 30 days of age and show increased levels of foraging behaviour at 42 days of age [27]. Since feather pecking starts much earlier than the differences in general exploratory and foraging behaviour (i.e., pecks directed at objects and materials other than conspecifics) between brooded and non-brooded chicks can be observed, it appears that increased injurious pecking in non-brooded birds does not result from exploratory and foraging behaviour per se but more likely because non-brooded chickens recognise feathers as an interesting material for exploration.

Active individuals are less likely to disturb the resting conspecifics if different areas are used for resting and active behaviours, as seems to be the case when dark brooders are provided. Indeed, dark brooders have been shown to improve rest in young chicks. In a study by Riber and Guzman [27], instantaneous observations were obtained during the light period of the day on the number of resting individuals in the first 4 days, as well as on days 9, 16, 23, 30 and 42 of age. Summed up per day, they found that a greater percentage of dark-brooded chicks rested compared to non-brooded chicks in the first 4 days and at 16 days of age, indicating that, during the daytime, brooded chicks had longer total rest durations. In broilers, birds reared with brooders had longer resting bouts, fewer disturbances of rest and longer bouts of activity between resting bouts, indicating a better quality of rest [28]. Interestingly, these effects remained until slaughter, i.e., 2 weeks after the removal of the brooders. Similarly, brooded layer chicks showed longer active and inactive phases during the first 2 weeks of life compared to their non-brooded conspecifics, as well as being more synchronised in their activity/rest, indicating that they were likely less disturbed while resting, though, in this study, rearing with brooders did not affect the total time spent active [25]. Whether the difference in duration of resting bouts and the synchronisation of activity remain into adulthood remains to be studied. As sleep and learning capabilities are linked, the observed superiority in solving a spatial learning task in broilers reared with brooders compared to non-brooded birds [28] may be a further indicator of their improved quality of rest, though the experiences of navigating around and under the brooders may also have affected the results. Newberry et al. [39] suggested that injurious pecking in adulthood may be more common in birds that showed less resting behaviour as pullets; thus, improved rest in chicks reared with dark brooders may be one of the contributing factors in reducing injurious pecking. Stress is related to injurious pecking [9], and disturbances during rest may be stressful to the birds, though this has not been studied. Additionally, to reduce the prevalence of injurious pecking once injurious pecking appears, dark brooders may offer a hiding place for possible victims of early injurious pecking, preventing the escalation of the problem in older birds. However, this has not been studied.

It appears that fearfulness may be related to injurious pecking, although the cause and effect seem unclear [20,40,41]. Thus, efforts should strive to find management initiatives that lead to reduced fearfulness in layers. Pullets and layers reared with dark brooders show reduced fearfulness during fear tests [27]. This was supported by observations in the home pens, where fewer brooded pullets showed fleeing behaviour in response to the arrival of a human observer at any age tested (i.e., from 4 to 26 weeks) than non-brooded pullets [27]. Thus, the reduced fearfulness of pullets and layers reared with brooders may further explain why dark brooders successfully reduce injurious pecking in laying hens. In broiler chickens reared with brooders, no difference in fearfulness was detected when the birds were tested at 3 weeks of age [26].

Reduced fearfulness and improved rest (i.e., fewer disturbances during rest) in birds reared with dark brooders would be expected to lead to reduced physiological stress reactions. Rearing chicks in a complex environment where dark brooders are also provided as part of the enrichment buffers against physiological stress responses under unpredictable stress situations compared to rearing in a simple environment [42]. However, due to possible confounding and/or additive effects of the enrichment factors (including an improved three-dimensional space use) in a complex environment on physiological stress reactions, it is not possible to infer the specific effect of dark brooders from this study. Studies that focused only on dark brooders found no differences between layer pullets or hens reared with or without brooders in the level of faecal corticosterone metabolite at 2 weeks of age [12] or in the feather corticosterone levels at 16 and 28 weeks of age [43]. However, these measures likely reflect basal hypothalamus—pituitary—adrenal axis activity rather than a response to acute or chronic stress, because the birds were not exposed to any stressful events prior to the sample collections. It would be valuable to know if dark brooders improve their ability to cope with stressful events that the chickens are likely to experience at various stages of life, such as water restriction for vaccinations, transition from the rearing facility to the laying facility, unpredictable loud sounds, etc.

There are other possible welfare benefits of rearing chicks with dark brooders that remain to be studied, including the reduced occurrence of panic behaviour and improved range use. Panic behaviour is related to fearfulness and may result in injuries [44] or pilling/smothering [45,46]. Research on the effects of dark brooders on panic behaviour is scarce. In one of the batches in a study by Gilani et al. [14], the non-brooded pullets suffered from a smothering event and experienced 11% total mortality. Smothering has not been reported in brooded pullets. Similarly, in a demonstration project on dark brooders, smothering occurred in one of the non-brooded flocks due to maintenance of the ventilation on the roof, resulting in high mortality [47]. Although the brooded birds were exposed to the same experience, it did not result in fatalities. The effects of dark brooders on smothering behaviour, as well as injuries related to panic behaviour, should be investigated further before any conclusions can be drawn.

Ranging behaviour may be positively affected by rearing with dark brooders. Pullets are reared indoors in the first weeks and only get outdoor access when older. It is known that the outdoor range in pullets and layers is not used to its full capacity. For example, one study showed that up to 31.4% of commercial layers do not range daily [48], and great effort is being made to increase the use of the range. Ranging behaviour may be influenced by the level of fearfulness in laying hens [49,50,51]. Laying hens that use the range most often are less fearful compared to hens that do not range [51]. Furthermore, it is also thought that increasing the explorative behaviour in pullets and layers could increase the use of a range [51]. As dark brooders decrease fearfulness and increase exploratory behaviour in pullets and layers, it is possible that rearing with dark brooders may positively influence the use of an outdoor range in laying hens. However, the only study on the effect of brooders on ranging behaviour done up until now was conducted on broiler chickens, where the effects of brooders were not found on fearfulness or on the range use. Thus, future research should investigate the effect of brooders on the range use in layers.

A final point to mention is the potential benefit that dark brooders have on the quality of rest. Disruptions of rest are detrimental to welfare and health across the taxa [52,53,54,55]. Studies in broiler chickens have shown that providing an extensive dark period (10 h compared with 1, 4 or 7 h) reduces mortality [55], and an increase in the daily period of darkness, across the range of 0–6.5 h, is associated with improved health and reduced fear of humans [56]. Thus, the improved sleep of brooded chicks may have welfare benefits beyond the reduction of injurious pecking, but this remains to be studied.

To sum up, dark brooders are advantageous for layer welfare during rearing and production. The main benefit of dark brooders is the prevention and/or reduction of the prevalence of injurious pecking [12,14], likely due to the separation of active and inactive individuals [13] and reduced fearfulness [27]. As a result, dark-brooded birds have improved plumage conditions [12,13,14], fewer skin injuries [12,13] and reduced mortality caused by cannibalism [12,13]. Reduced fearfulness could be linked to further welfare benefits, such as an improved range use and fewer injuries due to panic behaviour, but this remains to be confirmed.

## 5. Challenges to Overcome before Dark Brooders Are Widely Introduced in Commercial Rearing of Pullets

The reasons for a limited instalment of brooders in commercial facilities have not been studied, but we speculate that the lack of commercially available brooder options and the sizes of the brooders that may not fit inside the aviary systems, as well as a lack of information on the direct economic aspects, likely contribute to their low usage in commercial systems. In the following, we will discuss these factors.

Dark brooders show advantages beyond improved welfare and likely bring economic benefits, though a proper economic study has not yet been conducted. As a barn can be kept cooler, less energy is needed to heat them, resulting in direct reduced costs during the rearing period. Further benefits may come from improved production, as the total egg production increases when mortality is reduced [13]. Moreover, dark-brooded layers, as compared to non-brooded layers, housed in small groups use nests more readily, which leads to a reduction in floor eggs [13]. Egg production in relation to the use of dark brooders has only been studied in small flocks of brown genotypes so far. Thus, additional research on commercial farms with large flocks of either white or brown genotypes is needed before conclusions on the economic benefits for commercial egg producers can be drawn. The result of mortality due to other causes than injurious pecking (i.e., general mortality) are less clear. Gilani et al. [14] reported no effect of brooders on general mortality until 35 weeks of age in a commercial system, whereas Riber and Guzmán [13] reported a reduced general mortality in dark-brooded as compared to non-brooded birds during the laying period in a study conducted under experimental settings. This may reflect the high level of skin injuries in non-brooded birds found in the latter study, increasing the risk of secondary infections, but further research is needed to confirm this. The feed intake in relation to rearing pullets with dark brooders has not been studied so far. However, dark brooders may indirectly affect the feed intake and feed conversion ratio by reducing injurious pecking and improving the feather cover. The feed intake of hens with good feather cover has been shown to be 16% lower than for hens with poor feather cover, improving the feed conversion ratio [8]. It has been estimated that injurious pecking and associated plumage damage may result in an increase of 7–12% in the cost of egg production [8]. The possible downsides of dark brooders may be the cost of purchase, especially since the cost of brooders currently commercially available is relatively high, and available brooders may need to be adapted for commercial use (as discussed later). Dark brooders may also demand extra man hours for the inspection of chicks during the first few days to ensure the good use of brooders. However, this remains to be examined. Research focusing on the economic aspects, including the workload involved in operating dark brooders, may aid pullet producers in deciding whether to install brooders on their farms.

The sizes of the brooders may be regarded as an issue, as the instalment of brooders may leave insufficient space for other behavioural needs and access to feed and water. Space issues may be especially high inside the aviary systems where chicks spend up to the first 5 weeks of life before they are allowed to use the whole barn. Based on a study comparing the effect of large (72 cm^2^ per chick) and small (54 cm^2^ per chick) dark brooders, the development of injurious pecking did not seem to be influenced by the sizes of brooders [27]. Other welfare-related factors were also not influenced by the brooder size. For example, a similar number of chicks rested under the small and large brooders until 42 days of age, when the last behavioural observations were made [27]. It is possible that even smaller brooders would suffice, especially if the time period they are provided can be shortened. Planimetry, which considers the area occupied by the animal’s body, could be used to determine the minimum brooder size necessary. It has shown that the area occupied by pullets of various breeds has a linear relationship with their body weight [57], but only pullets older than 6 weeks of age have been studied so far. Smaller brooders, shorter provision and/or the use of unheated brooders would cut down on the purchase costs and reduce the workload of the farmers, but the effects on injurious pecking and other welfare-related factors should be examined, as the use of, e.g., non-heated brooders may limit the positive effects on welfare documented in studies with heated brooders. Another concern with respect to the sizes of dark brooders in aviary systems is that sufficient vertical space is required to enable adjustments of the brooders to the heights of the chicks and for daily inspection. Furthermore, the removal of dark brooders should not be labour-intensive or time-consuming. Ideally, once not in use, brooders should be lifted and left inside the barn to minimise disturbance of the flock.

There may also be concern that young chicks will not leave the brooders to search for feed and water. If this is the case, brooders would need to be regularly raised to encourage the chicks to become active and initiate feed and water consumption. However, research shows that this concern is unfounded. Comparing chicks reared with brooders that were raised multiple times a day or remained stationary revealed no differences in the feeding and drinking behaviour, body weight and mortality during the first week of life [13]. Thus, lifting dark brooders regularly may be skipped, leaving the design of brooders simple and cheap.

**Figure 3:**
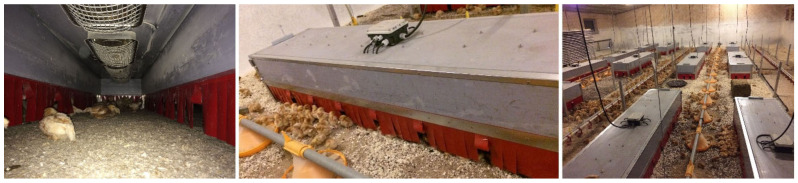
Home-built dark brooders in an organic commercial pullet farm. The brooders have a lid for the farmer to easily inspect the chicks underneath the brooder. Left: inside with heaters mounted on the ceiling, middle: outside with a temperature control system on the top and parts of the fabric fringes lifted to guide the chicks in and out of the brooder and right: barn with brooders positioned between the feeder and drinker lines. ©Tina Bøje Clausen.

The main problem in the low usage of dark brooders is probably that there are no commercial options for large-scale pullet rearing. While a single company advertises dark brooders on their website, they seem to be in the prototype phase (i.e., we were not able to purchase one). Thus, farmers are left on their own to build dark brooders and establish the best management practices (e.g., Figure 3).

Brooders that emit heat can be difficult to produce and regulate. Hence, some farmers provide only darkness, and although chickens still use those brooders to some extent, no research has been provided to show the effect of this kind of brooder on bird welfare and production. A brooder built by using small, already commercially available brooders may be a reasonable option for aviaries, as it offers great flexibility. However, these brooders need to be modified so that it is easier to manipulate them (e.g., connect them to larger units that can be lifted simultaneously), and fringes are usually missing in the commercially available options. Commercially available brooders are relatively flat and will—when mounted on the tier above in an aviary—only require a few centimetres during storage, leaving sufficient space underneath once they are fully lifted. The largest commercially available brooder we were able to find measures 40 × 60 cm, with the heating element (i.e., the roof) only approximately 3 cm high. A single brooder of these dimensions would thus provide 54 cm^2^/bird to 45 chicks or 48 cm^2^/bird to 50 chicks and would easily fit inside an aviary system. However, farmers are still left on their own to install and modify them.

The economic benefits are mainly found during the laying period, except the savings on heating costs that the pullet producers may gain by transitioning to brooders. In contrast, the costs associated with brooders are connected to the rearing period, including purchasing brooders and modifying and installing brooders, as well as the extra man hours for the inspection of chicks during the first few days. Thus, knowledge on the benefits of brooders should be disseminated not only to pullet producers but also to the egg producers for the latter to accept compensating the pullet producers by paying a premium for pullets reared with brooders.

Consumers and policymakers demand more sustainable production practices [58]. A system is viewed as sustainable if it has beneficial effects on human health, animal welfare or the environment [58]. At least two of the sustainability factors are improved when dark brooders are provided to chicks. Dark-brooded pullets and layers have improved welfare due to reduced injurious pecking [12,14]. Further, dark brooders likely to reduce the impact on the environment through the energy efficiency in the rearing phase and improved feed efficiency during the laying phase. Improved egg production [13], where fewer animals are needed to satisfy the demand for eggs, also likely contributes to the improved sustainability. However, future research will need to focus on the effects of brooders on the feed intake and feed conversion ratio to provide complete information on the sustainability of brooders.

## 6. Conclusions

Dark brooders are rarely used in commercial layer pullet facilities, despite their advantages for animal welfare and the likely positive outcomes in production and the economy. It is well-documented that dark brooders have a long-lasting reducing effect on injurious pecking, which improves feather cover and reduces mortality due to cannibalism. Consequently, feed consumption is likely reduced, and the total egg production is increased, influencing the production economy positively. One important gap of knowledge that remains is the effect of brooders on white genotypes of laying hens, as they differ from brown genotypes in many aspects, including the propensity to perform the different types of injurious pecking. The main limitation in the use of brooders in commercial facilities is likely the insufficient knowledge on the economic aspects of using brooders in commercial settings, including economic cost-benefit analyses and the lack of commercially available brooder options. Research is needed on these aspects, and the results should be disseminated broadly to ensure that end users are reached.

## Data Availability

Not applicable.

## References

[1] van Niekerk T. (2019). Evidence-Based Management of Injurious Pecking. Poult. Feathers Ski. Poult. Integument Heal. Welf..

[2] Savory C.J. (1995). Feather Pecking and Cannibalism. Worlds Poult. Sci. J..

[3] Gentle M.J. (2011). Pain Issues in Poultry. Appl. Anim. Behav. Sci..

[4] Fossum O., Jansson D.S., Etterlin P.E., Vågsholm I. (2009). Causes of Mortality in Laying Hens in Different Housing Systems in 2001 to 2004. Acta Vet. Scand..

[5] Bestman M., Verwer C., Brenninkmeyer C., Willett A., Hinrichsen L.K., Smajlhodzic F., Heerkens J.L.T., Gunnarsson S., Ferrante V. (2017). Feather-Pecking and Injurious Pecking in Organic Laying Hens in 107 Flocks from Eight European Countries. Anim. Welf..

[6] Gentle M.J., Hunter L.N. (1991). Physiological and Behavioural Responses Associated with Feather Removal in Gallus Gallus Var Domesticus. Res. Vet. Sci..

[7] Peguri A., Coon C. (1993). Effect of Feather Coverage and Temperature on Layer Performance. Poult. Sci..

[8] Glatz P.C. (2001). Effect of Poor Feather Cover on Feed Intake and Production of Aged Laying Hens. Asian-Australas. J. Anim. Sci..

[9] Cronin G.M., Glatz P.C. (2020). Causes of Feather Pecking and Subsequent Welfare Issues for the Laying Hen: A Review. Anim. Prod. Sci..

[10] Nicol C.J., Bestman M., Gilani A.M., De Haas E.N., De Jong I.C., Lambton S., Wagenaar J.P., Weeks C.A., Rodenburg T.B. (2013). The Prevention and Control of Feather Pecking: Application to Commercial Systems. Worlds. Poult. Sci. J..

[11] Janczak A.M., Riber A.B. (2015). Review of Rearing-Related Factors Affecting the Welfare of Laying Hens. Poult. Sci..

[12] Jensen A.B., Palme R., Forkman B. (2006). Effect of Brooders on Feather Pecking and Cannibalism in Domestic Fowl (*Gallus gallus domesticus*). Appl. Anim. Behav. Sci..

[13] Riber A.B., Guzmán D.A. (2017). Effects of Different Types of Dark Brooders on Injurious Pecking Damage and Production-Related Traits at Rear and Lay in Layers. Poult. Sci..

[14] Gilani A.-M., Knowles T.G., Nicol C.J. (2012). The Effect of Dark Brooders on Feather Pecking on Commercial Farms. Appl. Anim. Behav. Sci..

[15] Sherry D.F. (1981). Parental Care and the Development of Thermoregulation in Red Junglefowl. Behaviour.

[16] Edgar J., Held S., Jones C., Troisi C. (2016). Influences of Maternal Care on Chicken Welfare. Animals.

[17] UKAG, Extension, (University of Kentucky) Poultry Production Manual. https://afs.ca.uky.edu/poultry/production-manual.

[18] Fält B. (1978). Differences in Aggressiveness between Brooded and Non-Brooded Domestic Chicks. Appl. Anim. Ethol..

[19] Pittet F., Le Bot O., Houdelier C., Richard-Yris M., Lumineau S. (2014). Motherless Quail Mothers Display Impaired Maternal Behavior and Produce More Fearful and Less Socially Motivated Offspring. Dev. Psychobiol..

[20] Rodenburg T.B., Buitenhuis A.J., Ask B., Uitdehaag K.A., Koene P., Van Der Poel J.J., Van Arendonk J.A.M., Bovenhuis H. (2004). Genetic and Phenotypic Correlations between Feather Pecking and Open-Field Response in Laying Hens at Two Different Ages. Behav. Genet..

[21] Riber A.B., Wichman A., Braastad B.O., Forkman B. (2007). Effects of Broody Hens on Perch Use, Ground Pecking, Feather Pecking and Cannibalism in Domestic Fowl (*Gallus gallus domesticus*). Appl. Anim. Behav. Sci..

[22] Roden C., Wechsler B. (1998). A Comparison of the Behaviour of Domestic Chicks Reared with or without a Hen in Enriched Pens. Appl. Anim. Behav. Sci..

[23] Rodenburg T.B., Uitdehaag K.A., Ellen E.D., Komen J. (2009). The Effects of Selection on Low Mortality and Brooding by a Mother Hen on Open-Field Response, Feather Pecking and Cannibalism in Laying Hens. Anim. Welf..

[24] Perré Y., Wauters A.-M., Richard-Yris M.-A. (2002). Influence of Mothering on Emotional and Social Reactivity of Domestic Pullets. Appl. Anim. Behav. Sci..

[25] Riber A.B., Nielsen B.L., Ritz C., Forkman B. (2007). Diurnal Activity Cycles and Synchrony in Layer Hen Chicks (*Gallus gallus domesticus*). Appl. Anim. Behav. Sci..

[26] Stadig L.M., Rodenburg T.B., Reubens B., Ampe B., Tuyttens F.A.M. (2018). Effects of Dark Brooders and Overhangs on Free-Range Use and Behaviour of Slow-Growing Broilers. Animal.

[27] Riber A.B., Guzmán D.A. (2016). Effects of Dark Brooders on Behavior and Fearfulness in Layers. Animals.

[28] Forslind S., Wall H., Hernandes C.E., Riber A.B., Wall H., Blokhuis H.J. (2022). Resting Behaviour of Broilers Reared with or without Artificial Brooders. Front. Vet. Sci..

[29] Buitenhuis A.J., Rodenburg T.B., Siwek M., Cornelissen S.J.B., Nieuwland M.G.B., Crooijmans R., Groenen M.A.M., Koene P., Bovenhuis H., Van Der Poel J.J. (2005). Quantitative Trait Loci for Behavioural Traits in Chickens. Livest. Prod. Sci..

[30] Lambton S.L., Knowles T.G., Yorke C., Nicol C.J. (2015). The Risk Factors Affecting the Development of Vent Pecking and Cannibalism in Free-Range and Organic Laying Hens. Anim. Welf..

[31] Rodenburg T.B., Van Hierden Y.M., Buitenhuis A.J., Riedstra B., Koene P., Korte S.M., Van Der Poel J.J., Groothuis T.G.G., Blokhuis H.J. (2004). Feather Pecking in Laying Hens: New Insights and Directions for Research?. Appl. Anim. Behav. Sci..

[32] Blokhuis H.J., Arkes J.G. (1984). Some Observations on the Development of Feather-Pecking in Poultry. Appl. Anim. Behav. Sci..

[33] Hess E.H. (1964). Imprinting in Birds. Science.

[34] Sanotra G.S., Vestergaard K.S., Agger J.F., Lawson L.G. (1995). The Relative Preferences for Feathers, Straw, Wood-Shavings and Sand for Dustbathing, Pecking and Scratching in Domestic Chicks. Appl. Anim. Behav. Sci..

[35] Brown L.T. (1964). A Critical Period in the Learning of Motionless Stimulus Properties in Chicks. Anim. Behav..

[36] Vestergaard K.S., Baranyiova E. (1996). Pecking and Scratching in the Development of Dust Perception in Young Chicks. Acta Vet. Brno.

[37] Blokhuis H.J. (1986). Feather-Pecking in Poultry: Its Relation with Ground-Pecking. Appl. Anim. Behav. Sci..

[38] Riber A.B., Forkman B. (2007). A Note on the Behaviour of the Chicken That Receives Feather Pecks. Appl. Anim. Behav. Sci..

[39] Newberry R.C., Keeling L.J., Estevez I., Bilčík B. (2007). Behaviour When Young as a Predictor of Severe Feather Pecking in Adult Laying Hens: The Redirected Foraging Hypothesis Revisited. Appl. Anim. Behav. Sci..

[40] Jensen P., Keeling L., Schütz K., Andersson L., Mormède P., Brändström H., Forkman B., Kerje S., Fredriksson R., Ohlsson C. (2005). Feather Pecking in Chickens Is Genetically Related to Behavioural and Developmental Traits. Physiol. Behav..

[41] Jones R.B., Blokhuis H.J., Beuving G. (1995). Open-field and Tonic Immobility Responses in Domestic Chicks of Two Genetic Lines Differing in Their Propensity to Feather Peck. Br. Poult. Sci..

[42] Campderrich I., Nazar F.N., Wichman A., Marin R.H., Estevez I., Keeling L.J. (2019). Environmental Complexity: A Buffer against Stress in the Domestic Chick. PLoS ONE.

[43] Nordquist R.E., Zeinstra E.C., Dougherty A., Riber A.B. (2020). Effects of Dark Brooder Rearing and Age on Hypothalamic Vasotocin and Feather Corticosterone Levels in Laying Hens. Front. Vet. Sci..

[44] Harlander-Matauschek A., Rodenburg T.B., Sandilands V., Tobalske B.W., Toscano M.J. (2015). Causes of Keel Bone Damage and Their Solutions in Laying Hens. Worlds Poult. Sci. J..

[45] Gray H., Davies R., Bright A., Rayner A., Asher L. (2020). Why Do Hens Pile? Hypothesizing the Causes and Consequences. Front. Vet. Sci..

[46] Richards G.J., Brown S.N., Booth F., Toscano M.J., Wilkins L.J. (2012). Panic in Free-Range Laying Hens. Vet. Rec..

[47] Clausen T.B. (2019). Bedre Fra Start Med Kunstige Kyllingemødre—Vejledning Til Og Erfaringer Med Anvendelse Af Kunstige Kyllingemødre i Opdrætsperioden Hos Gulvopdræt Af Økologiske Æglæggende Høner. [In Danish: Better Start-up with Dark Brooders—A Guide to and Experience.

[48] Larsen H., Cronin G.M., Gebhardt-Henrich S.G., Smith C.L., Hemsworth P.H., Rault J.-L. (2017). Individual Ranging Behaviour Patterns in Commercial Free-Range Layers as Observed through RFID Tracking. Animals.

[49] Grigor P.N., Hughes B.O., Appleby M.C. (1995). Effects of Regular Handling and Exposure to an Outside Area on Subsequent Fearfulness and Dispersal in Domestic Hens. Appl. Anim. Behav. Sci..

[50] Hartcher K.M., Hickey K.A., Hemsworth P.H., Cronin G.M., Wilkinson S.J., Singh M. (2016). Relationships between Range Access as Monitored by Radio Frequency Identification Technology, Fearfulness, and Plumage Damage in Free-Range Laying Hens. Animal.

[51] Bari M.S., Allen S.S., Mesken J., Cohen-Barnhouse A.M., Campbell D.L.M. (2021). Relationship between Range Use and Fearfulness in Free-Range Hens from Different Rearing Enrichments. Animals.

[52] Abou-Ismail U.A., Burman O.H.P., Nicol C.J., Mendl M. (2008). Let Sleeping Rats Lie: Does the Timing of Husbandry Procedures Affect Laboratory Rat Behaviour, Physiology and Welfare?. Appl. Anim. Behav. Sci..

[53] Diverio S., Tami G., Barone A. (2008). Prevalence of Aggression and Fear-Related Behavioral Problems in a Sample of Argentine Dogos in Italy. J. Vet. Behav..

[54] Opp M.R., Krueger J.M. (2015). Sleep and Immunity: A Growing Field with Clinical Impact. Brain. Behav. Immun..

[55] Schwean-Lardner K., Fancher B.I., Classen H.L. (2012). Impact of Daylength on the Productivity of Two Commercial Broiler Strains. Br. Poult. Sci..

[56] Bassler A.W., Arnould C., Butterworth A., Colin L., De Jong I.C., Ferrante V., Ferrari P., Haslam S., Wemelsfelder F., Blokhuis H.J. (2013). Potential Risk Factors Associated with Contact Dermatitis, Lameness, Negative Emotional State, and Fear of Humans in Broiler Chicken Flocks. Poult. Sci..

[57] Spindler B., Clauss M., Briese A., Hartung J. (2013). Planimetric Measurement of Floor Space Covered by Pullets. Berl. Munch. Tierarztl. Wochenschr..

[58] Broom D.M., Galindo F.A., Murgueitio E. (2013). Sustainable, Efficient Livestock Production with High Biodiversity and Good Welfare for Animals. Proc. R. Soc. B Biol. Sci..

